# Infections among Contacts of Patients with Nipah Virus, India

**DOI:** 10.3201/eid2505.181352

**Published:** 2019-05

**Authors:** C.P. Girish Kumar, Attayur P. Sugunan, Pragya Yadav, Karishma Krishna Kurup, Renjith Aarathee, Ponnaiah Manickam, Tarun Bhatnagar, Chandni Radhakrishnan, Beena Thomas, Akhilesh Kumar, Jayasree Jayasree, Bina Philomina, K.G. Sajeeth Kumar, N.K. Thulaseedharan, Nivedita Gupta, R. Rajendran, R.L. Saritha, Devendra T. Mourya, Raman R. Gangakhedkar, Manoj V. Murhekar

**Affiliations:** ICMR National Institute of Epidemiology, Chennai, India (C.P.G. Kumar, K.K. Kurup, R. Aarathee, P. Manickam, T. Bhatnagar, M.V. Murhekar);; ICMR–Regional Medical Research Centre, Port Blair, India (A.P. Sugunan);; ICMR–National Institute of Virology, Pune, India (P. Yadav, D.T. Mourya);; Directorate of Health Services, Government of Kerala, Kerala (R. Aarathee, A. Kumar, V. Jayasree, R.L. Saritha);; Government Medical College, Kozikhode, India (C. Radhakrishnan, B. Thomas, B. Philomina, K.G.S. Kumar, N.K. Thulaseedharan, V.R. Rajendran);; Indian Council of Medical Research, New Delhi, India (N. Gupta, R.R. Gangakhedkar)

**Keywords:** contact tracing, healthcare workers, India, Nipah virus, viruses, subclinical infection

## Abstract

We conducted a serosurvey of 155 healthcare workers and 124 household and community members who had close contact with 18 patients who had laboratory-confirmed Nipah virus infections in Kerala, India. We detected 3 subclinical infections; 2 persons had IgM and IgG and 1 only IgM against Nipah virus.

Nipah virus (NiV) infection is an emerging zoonotic disease that has the potential to cause severe disease in both animals and humans (*1*). Fruit bats of the *Pteropus* genus (family *Pteropodidae*) are the natural hosts of NiV (*2*). Outbreaks of NiV have been reported from Malaysia, Singapore, Bangladesh, and eastern India; mortality rates are 40%–70% (*3*–*5*). In an outbreak in Malaysia, pigs were intermediate hosts and most human infections occurred from close contact with infected pigs (*6*), whereas during outbreaks in Bangladesh, ingestion of date palm sap contaminated with saliva or excreta from infected fruit bats was the main spillover route (*7*). During outbreaks in Bangladesh (*4*) and West Bengal, India (*5*), person-to-person transmission occurred among close contacts, including healthcare workers (HCWs), after initial spillover of NiV into humans.

During May 2018, an NiV outbreak occurred in the Kozhikode and Malappuram districts of Kerala, India (*8*). The initial case-patient was hospitalized on May 3, 2018, but his blood sample could not be collected for laboratory confirmation of NiV. During May 3–29, NiV infection was confirmed in another 18 patients, linked to the initial probable case-patient, through detection of NiV RNA by reverse transcription PCR of throat swab, urine, or blood samples. Sixteen patients with laboratory-confirmed NiV infection died (case-fatality rate 89%). Although the source of infection for the initial case remained unknown, all subsequent cases occurred by person-to-person transmission through close contact with NiV patients.

As part of contact tracing, district health authorities identified ≈2,600 contacts of laboratory-confirmed NiV patients. Contacts were classified into 5 categories depending on the type of exposure they had with patients, similar to the scheme of classification followed during Ebola outbreaks (*9*). Contacts were monitored for 21 days postexposure for development of febrile illness.

Although 17 of the 18 laboratory-confirmed NiV patients exhibited acute neurologic or respiratory symptoms, 1 had mild, uncomplicated febrile illness. This patient had a history of close contact with another laboratory-confirmed patient and survived after being treated with ribavirin and supportive therapy. Laboratory-confirmed infection in a NiV patient with only mild febrile illness raised a question of whether additional, mildly symptomatic or asymptomatic NiV infections might also occur among close contacts in this outbreak. To address this question, we conducted a cross-sectional study during July 2–13, 2018 (60–71 days after the initial case was hospitalized), of persons with high-risk exposure to NiV patients to estimate the seroprevalence of NiV-specific IgM and IgG.

## The Study

We used a line list of high-risk exposure contacts of the initial 18 laboratory-confirmed NiV patients, including 235 HCWs and 191 household and community contacts. We needed to survey 300 contacts (150 HCW and 150 household and community contacts) because our assumption was that 5% of contacts would have asymptomatic infection de-velop, and absolute precision of the estimate would be 2.5% for a 95% confidence level. The Institutional Human Ethics Committee of the ICMR–National Institute of Epidemiology, Chennai (approval no. NIE/IHEC/201806-01) and the Government Medical College, Kozhikode (approval no. GMCKKD/RP2018/IEC/97) approved the study protocol.

We approached the contacts at their residences or workplaces and interviewed them using a structured questionnaire. We collected sociodemographic information, data on the type and frequency of contact with >1 NiV patient, and history of febrile illness after contact with NiV patients. For each person who consented to participate, we collected a 3-mL blood sample, separated serum, and transported samples to the National Institute of Virology (Pune, India), where they were tested for human IgM and IgG against NiV. 

We collected 279 blood samples from 155 HCWs and 124 household and community contacts. The median age for HCWs was 37 years (interquartile range 29–48 years) and for household and community contacts was 39 years (interquartile range 30–51 years). Thirty-two HCWs and 36 household contacts reported exposure to body fluids of NiV patients; 123 (79.4%) HCWs and 88 (71.0%) household contacts reported physical contact with ≥1 NiV patient (Table 1).

**Table 1:** Distribution of contacts of patients with laboratory-confirmed Nipah virus by selected characteristics, Kerala, India, 2018

Characteristic	Healthcare workers, no. (%), n = 155	Household and community contacts, no. (%), n = 124
Age, y		
<15	0	3 (2.4)
15–45	103 (66.4)	72 (58.1)
>45	52 (33.6)	49 (39.5)
Sex		
M	39 (25.2)	91 (73.4)
F	116 (74.8)	33 (26.6)
Type of exposure		
In patient’s room	123 (79.4)	113 (91.1)
Contact with patient, no contact with body fluids	123 (79.4)	88 (71.0)
Exposure to body fluids*	32 (20.6)	36 (29.0)
Saliva	5 (3.2)	28 (22.6)
Cough	15 (9.7)	16 (12.9)
Vomit	6 (3.9)	14 (11.3)
Blood	10 (6.5)	0

*Contacts reported exposure to >1 type of body fluid.

We performed ELISA on samples with reagents provided by the US Centers for Disease Control and Prevention (Atlanta, GA, USA) and tested serum at 4 dilutions: 1:100, 1:400, 1:1,600, and 1:6,400. For IgM assays, we considered samples positive when the sum of the optical density for all 4 dilutions was >0.45 (*10*). For IgG assays, we considered samples positive when the sum of the optical density for all dilutions was >0.95. 

Of the 279 serum samples tested, 2 had IgM and IgG and 1 had only IgM against NiV. We calculated the overall seroprevalence of NiV as 1.08% (95% CI 0.37–3.11). None of the seropositive persons reported having a febrile illness after their last contact with an NiV patient, indicating subclinical infections. Two seropositive persons were family members of a laboratory-confirmed patient, and the third was a HCW in the emergency medicine department. All 3 had a history of exposure to body fluids of >1 NiV patient (Table 2).

**Table 2:** Details of Nipah virus seropositive samples among close patient contacts, Kerala, India, 2018

Characteristic	Contact 1*	Contact 2*	Contact 3
Age, y	58	60	49
Sex	M	F	M
Date of contact	22 May 2018	22 May 2018	5 May 2018
Time between exposure and blood sample collection	49 d	49 d	63 d
Laboratory findings			
IgM against Nipah virus (optical density)	Positive (0.55)	Positive (0.77)	Positive (0.46)
IgG against Nipah virus (optical density)	Positive (0.85)	Positive (1.86)	Negative (0.09)
Time of exposure			
During patient’s illness	Yes	Yes	Yes
On day of patient’s death	Yes	Yes	No
After patient’s death (e.g., funeral rituals)	Yes	Yes	No
Type of exposure			
Touched patient	Yes	Yes	Yes
Had contact with a patient’s body fluids	Yes	Yes	Yes
Spent >1 day with a patient in same room or ward	Yes	Yes	No
Fed patient with hands	No	Yes	No
Changed patient’s bed linen	No	Yes	No
Changed patient’s clothes	No	Yes	No
Washed patient’s bed linen	No	Yes	No
Washed patient’s clothes	No	Yes	No

*Family member of patient with Nipah virus.

The risk for subclinical infection was higher among the contacts who had exposure to body fluids (3/68, 4.4% [95% CI 1.5%–12.2%]) than for those who only had physical contact with ≥1 NiV patient (0/211, 0% [95% CI 0%–1.8%]; p = 0.007). The epidemiologic association between exposure and seropositivity suggests our results are accurate. Applying the proportion of asymptomatic infection found in our sample of 279 to all 426 persons exposed to laboratory-confirmed NiV infection yields an expected total of 23 NiV infections among contacts, including 5 (21.7%) asymptomatic cases.

## Conclusions

Although NiV is known to cause subclinical infections, the extent of these infections among close contacts varies during outbreaks. For instance, no subclinical infections have been reported from outbreaks in Bangladesh (*11*), but 1%–15% of infections were subclinical during outbreaks in Malaysia (*12*–*15*). Parashar et al. reported clinically undetected NiV infection in 6% of 166 community-farm controls and in 11% of 178 case-farm controls (*12*). Another study of household contacts of hospitalized NiV patients indicated that 8% had subclinical infections (*13*). In an outbreak in Singapore, infections were reported in 2 (4.6%) of 43 asymptomatic abattoir workers (*14*). Another study conducted in Singapore among 1,460 HCWs having contact with NiV patients identified antibodies specific for NiV in 22 (1.5%), 10 of whom were asymptomatic (*15*). These studies suggest that infection with the Malaysian strain of NiV causes less severe illness, a lower case-fatality rate, and higher prevalence of asymptomatic infections compared with outbreaks involving the Bangladesh strain. Studies in African green monkeys also suggest the Bangladesh strain of NiV is more pathogenic than the Malaysian strain (*1*). The NiV strain responsible for the Kerala outbreak was closer to the Bangladesh strain and was more pathogenic (*8*). Although previous studies did not show any subclinical infections during NiV outbreaks with the Bangladesh strain, our study suggested that NiV strain of Kerala outbreak generated asymptomatic infections. Our study also found that IgM could be detected ≤2 months after NiV infection and the immunoglobulin class switch to IgG could occur beyond 2 months.

Our study had 1 limitation. Although we approached all line listed contacts, we collected samples from only 124 of 191 household and community members. The remaining contacts were either unavailable (17%) or declined to give a blood sample (18%). However, this limitation is unlikely to affect overall seroprevalence because nonparticipation in the survey was not based on exposure status.

Our findings indicate that subclinical infections occurred among close contacts of patients during an NiV outbreak in Kerala, India, but were infrequent. In addition, we found the risk for subclinical infections was higher among persons with a history of exposure to body fluids of NiV patients than for those with only physical contact.
